# Is a Mass Immunization Program for Pandemic (H1N1) 2009 Good Value for Money? Early Evidence from the Canadian Experience.

**DOI:** 10.1371/currents.RRN1137

**Published:** 2009-12-17

**Authors:** Beate Sander, Chris Bauch, David N. Fisman, R Fowler, Jeffrey C. Kwong, Allison McGeer, Marija Zivkovic Gojovic, Murray Krahn

**Affiliations:** ^*^Department of Health Policy, Management and Evaluation, University of Toronto, Toronto, Canada; ^†^University of Guelph; ^‡^Dalla Lana School of Public Health, University of Toronto; ^§^Sunnybrook Hospital, The University of Toronto; ^¶^Institute for Clinical Evaluative Sciences, Toronto, Ontario, Canada; ^#^Department of Laboratory Medicine and Pathobiology, University of Toronto; ^**^Toronto Health Economics and Technology Assessment Collaborative(THETA) and ^††^University of Toronto

## Abstract

This work contributes informed estimates to the current debate about the pandemic (H1N1) 2009 mass immunization program’s economic merits. We performed a cost-utility analysis of the (H1N1) 2009 mass immunization program in Ontario, Canada’s most populous province. The analysis is based on a simulation model of a pandemic (H1N1) 2009 outbreak, surveillance data, and administrative data. We consider no immunization versus mass immunization reaching 30% of the population. Immunization program costs are expected to be $118 million in Ontario. Our analysis indicates this program will reduce influenza cases by 50%, preventing 35 deaths, and cutting treatment costs in half. A pandemic (H1N1) 2009 immunization program is likely to be highly cost-effective.

## Background

Since the H1N1 vaccine approval on October 21, 2009 in Canada, the largest vaccination program in the country’s history has been rolled out. The vaccine’s efficacy and the program’s effectiveness [1] have been estimated. However, in the face of rising vaccination program costs, there is increasing public discussion about whether the program represents good value for the healthcare dollar.

## Methods 

We performed a cost-utility analysis from the health care payer perspective perspective in the Canadian province of Ontario (population 13,000,000). This economic evaluation utilized a simulation model of a pandemic (H1N1) 2009 outbreak in a Canada city [2]. Attack rates for symptomatic cases were projected for four age groups under two strategies: (a) no vaccination, and (b) mass vaccination of 10% of the population per week, starting 40 days into the pandemic and lasting until 30% vaccine coverage is reached. We assume it takes 20 days for an individual to develop immunity. Patients with influenza A (H1N1) were treated with oseltamivir. Population-weighted mean predicted attack rates were 21% and 11% for strategies (a) and (b) respectively. Probabilities for health care resource use (office visits, emergency department (ED) visits, hospitalizations, intensive care unit (ICU) admissions, use of extracorporeal membrane oxygenation (ECMO)) and deaths were based on pandemic (H1N1) surveillance data in Ontario and Australia, and Ontario administrative data, covering the entire population of Canada’s most populous province. Hospitalization rates for Ontario were estimated from laboratory-confirmed cases, likely representing a significant underestimate of the true burden of disease. We therefore inflated reported rates 100-fold to account for expected underrepresentation of less severe H1N1 cases among laboratory-confirmed cases. This is consistent with estimates from the United States [3] and produces conservative predictions for number of hospitalizations, ICU admissions and deaths. Program and other costs were drawn from Ontario sources (Ontario Health Insurance Plan (OHIP), Ontario Case Costing Initiative (OCCI) [4]). Utility weights were obtained from the literature [5] and annualized. Years of life lost were calculated using average life expectancy adjusted for quality of life [6].

Main outcome measures were quality adjusted life-years (QALYs), costs in 2009 Canadian dollars, and cost per QALY gained.

**Table 1:** Predicted pandemic (H1N1) 2009 attack rates in Ontario [2]

**Table:** 

**Age Group**	**No Intervention**	**Immunization program covering 30% of the population starting 40 days into the pandemic**
0-4 years	0.25	0.12
5-19 years	0.39	0.22
20-64 years	0.17	0.08
65+ years	0.15	0.07
Weighted mean	0.21	0.11

**Table 2:** Pandemic (H1N1) 2009 Related Events

**Table:** 

**Event**	**Value**	**Source**
Office Visits^(a)^		50% of visits for seasonal influenza [OHIP]
0-4 years	0.39	
5-19 years	0.39	
20-64 years	0.39	
65+ years	0.39	
ED Visits^(b)^		
0-4 years	0.10	50% of visits for seasonal influenza [OHIP]
5-19 years	0.10	
20-64 years	0.10	
65+ years	0.10	
Hospitalization		Ontario H1N1 Spring Wave, denominator adjusted by 1/100 (100 symptomatic case per laboratory-confirmed case) [7]
0-4 years	0.00221	
5-19 years	0.00044	
20-64 years	0.00083	
65+ years	0.00325	
ICU Admission (non-ECMO) if hospitalized^(c)^		Australia H1N1 Data [8]
0-4 years	0.03	
5-19 years	0.05	
20-64 years	0.18	
65+ years	0.16	
ECMO if in ICU		Kumar A et al 2009 [9]
0-4 years	0.042	
5-19 years	0.042	
20-64 years	0.042	
65+ years	0.042	
Death per Hospitalized Case^(d)^		Australia H1N1 Data [8]
0-4 years	0.00532	
5-19 years	0.01144	
20-64 years	0.04424	
65+ years	0.09059	

Note: (a) 1.12 office visits per person (b) 1.17 Emergency Department visits per person (c) overall probability for ICU admission in Australia 0.13, in Ontario for the period August 30 to November 7, 2009: 0.12 (no age-specific rates available); (d) overall probability of death if hospitalized in Australia: 0.038, in Ontario for the period August 30 to November 7, 2009: 0.036 (no age-specific rates available); ED = emergency department; ICU = intensive care unit; ECMO = extracorporeal membrane oxygenation; OHIP = Ontario Health Insurance Plan; OCCI = Ontario Case Costing Initiative

**Table 3:** Pandemic (H1N1) 2009 Economic Data

**Table:** 

**Event**	**Value**	**Source**
Quality of life (annualized)		
No Influenza	1.0	Assumption
Symptomatic influenza		Turner D 2003 [5]
0-4 years	0.9854	
5-19 years	0.9854	
20-64 years	0.9826	
65+ years	0.9707	
Death	0.5	Assumption
Life years lost (undiscounted)		Statistics Canada 2008 [10], Mittmann N 1999 [6]
0-4 years	71.60	
5-19 years	62.26	
20-64 years	34.44	
65+ years	5.07	
Unit Cost (C$)		
Immunization per dose	30	Waldie P, 2009 [11]
Office visit	35	OHIP (seasonal influenza)
ED visit	220	OHIP (seasonal influenza) JPPC for non-physician costs (A9)
Hospitalization (non-ICU)		OCCI data 2007/2008 [4]
0-4 years	4,265	
5-19 years	4,265	
20-64 years	4,016	
65+ years	4,016	
ICU (non-ECMO)		OCCI data 2007/2008 [4]
0-4 years	14,350	
5-19 years	14,350	
20-64 years	11,676	
65+ years	11,676	
ICU+ECMO	142,132	OCCI data 2007/2008 [4]

ED = emergency department; ICU = intensive care unit; ECMO = extracorporeal membrane oxygenation; OHIP = Ontario Health Insurance Plan; OCCI = Ontario Case Costing Initiative

## Results

Ontario’s H1N1 immunization program is estimated to cost $118 million ($30 per person vaccinated). Immunizing 30% of the population prevents approximately 1.4 million cases, 850 hospitalizations and 35 deaths. This reduces healthcare cost due to illness from $154 million to $77 million and is associated with 24,864 additional quality-adjusted life-years for the population. The incremental cost-effectiveness ratio (ICER) is $1,645 per QALY gained. Results are sensitive to immunization program effectiveness and cost. If the program reduces the number of cases by only 25%, the ICER increases to $6,333 per QALY gained. Finally, if immunization costs are $50 per person vaccinated, the ICER compared to no intervention increases to $4,798 per QALY gained for 30% vaccination coverage. In all sensitivity analyses the ICER remains well below established thresholds, which determine the cost-effectiveness of a program [12].

**Table 4: **Aggregated Results for Base Case Analysis

**Table:** 

** **	**No Intervention** **Expected**	**30% Coverage** **Expected**	**30% Coverage** **Incremental**
Number Immunized	0	3,920,755	3,920,755
Cases	2,785,259	1,399,598	-1,385,661
Deaths	67	32	-35
Resource Use (Frequency)			
Office visits	1,201,004	603,507	-597,497
ED Visits	309,582	155,565	-154,016
Hospitalizations (All)	1,670	817	-853
Ward	1,449	711	-738
ICU	212	101	-110
ICU+ECMO	9	4	-5
Cost (C$)			
Immunization	0	117,622,638	117,622,638
Office visits	68,843,252	34,593,856	-34,249,395
ED Visits	74,722,934	37,548,407	-37,174,527
Hospitalizations (All)	10,333,022	5,025,517	-5,307,506
Ward	6,485,502	3,180,954	-3,304,548
ICU	2,528,428	1,213,146	-1,315,282
ICU+ECMO	1,319,093	631,416	-687,676
Total Health Care	153,899,207	77,167,780	-76,731,427
Total Cost	153,899,207	194,790,418	40,891,211
QALYs	124,932,891	124,957,756	24,864

ED = emergency department; ICU = intensive care unit; ECMO = extracorporeal membrane oxygenation; QALY = quality-adjusted life year

**Table 5:** Aggregated Results for Sensitivity Analyses

**Table:** 

	**No Intervention** **Expected**	**30% Coverage** **Expected**	**30% Coverage** **Incremental**
*Base Case*			
Cases	2,785,259	1,399,598	-1,385,661
Hospitalizations	1,670	817	-853
Deaths	67	32	-35
Total Cost	153,899,207	194,790,418	40,891,211
QALYs (undiscounted)	124,932,891	124,957,756	24,864
ICER (Cost/QALY)			1,645
*Sensitivity Analysis: Ratio 1/60*			
Cases	2,785,259	1,399,598	-1,385,661
Hospitalizations	2,783	1,362	-1,421
Deaths	111	53	-58
Total Cost	160,787,889	198,140,762	37,352,874
QALYs (undiscounted)	124,932,053	124,957,349	25,296
ICER (Cost/QALY)			1,477
*Sensitivity Analysis: Ratio 1/200*			
Cases	2,785,259	1,399,598	-1,385,661
Hospitalizations	835	409	-426
Deaths	33	16	-17
Total Cost	148,732,696	192,277,660	43,544,964
QALYs (undiscounted)	124,933,520	124,958,061	24,541
ICER (Cost/QALY)			1,774
*Sensitivity Analysis: lower vaccine program effectiveness*			
Cases	2,785,259	2,099,397	-685,863
Hospitalizations	1,670	1,226	-444
Deaths	67	48	-19
Total Cost	153,899,207	233,374,308	79,475,101
QALYs (undiscounted)	124,932,891	124,945,440	12,549
ICER (Cost/QALY)			6,333
*Sensitivity Analysis: high vaccination cost ($50 per person)*			
Cases	2,785,259	1,399,598	-1,385,661
Hospitalizations	1,670	817	-853
Deaths	67	32	-35
Total Cost	153,899,207	273,205,510	119,306,303
QALYs (undiscounted)	124,932,891	124,957,756	24,864
ICER (Cost/QALY)			4,798

ICER = Incremental cost-effectiveness ratio

## Discussion

We estimate that the pandemic (H1N1) 2009 mass immunization program in Ontario, Canada is highly cost-effective under conservative assumptions on healthcare resource use, costs, and mortality. This is consistent with the economic attractiveness demonstrated for seasonal influenza programs [13] [14].

Estimates of hospitalizations and mortality are based on laboratory-confirmed cases. In the base case, we expect 67 deaths. By contrast, the average annual estimated number of deaths related to seasonal influenza in Ontario is 500 to 1,200 ([15], and J.C. Kwong, unpublished). While the number of deaths associated with the 2009 pandemic may be lower in part because of relative sparing of older adults from disease, counting only laboratory-confirmed cases may also cause a significant underestimation. Similarly, we only include costs directly related to the treatment of pandemic (H1N1) cases. For example, the effects of the increased demand on health care services--such as cancelling elective surgery--are not included. Also, because of limited data this analysis also does not include vaccine-associated adverse events. While inclusion of serious adverse events reduces cost-effectiveness, we would not expect it to diminish the fundamental cost-effectiveness of the program if the incidence of such events remains low [2].

Public health physicians and officials have traditionally focused on containing the spread of infectious disease and mitigating disease burden. But those who organize and deliver public health services are also faced with the resource constraints that affect every other part of the healthcare system. We think it is unlikely that economic considerations will ever be, or should ever be uppermost in the minds of those battling a pandemic. However, it is reassuring to know that Canada's response to the current pandemic appears to be reasonable when viewed through the lens of careful stewardship of scarce resources. 

## Funding information

This study was supported by an operating grant from the Canadian Institutes for Health Research (CIHR), which provided fellowship support for B. Sander and M. Zivkovic Gojovic. B. Sander also received post-doc funding from MITACS (*Mathematics* of Information Technology and Complex Systems). D. Fisman holds an Ontario Early Researcher Award funded by the Ontario Ministry of Research and Innovation. R.A. Fowler holds a Clinician Scientist Award from the Ontario Ministry of Health and Long-term Care. J.C. Kwong holds a Career Scientist Award from the Ontario Ministry of Health and Long-term Care and a Research Scholar Award from the Department of Family and Community Medicine, University of Toronto. M. Krahn holds the F. Norman Hughes Chair in Pharmacoeconomics at the Faculty of Pharmacy, University of Toronto. Funders had no role in the design and conduct of the study; collection, management, analysis, and interpretation of the data; or preparation, review, or approval of the manuscript. The researchers are independent from the funders. 

This study was also supported by the Institute for Clinical Evaluative Sciences (ICES), which is funded by an annual grant from the Ontario Ministry of Health and Long-Term Care (MOHLTC). The opinions, results and conclusions reported in this paper are those of the authors and are independent from the funding sources. No endorsement by ICES or the Ontario MOHLTC is intended or should be inferred.

## Competing interests

Beate Sander has held consulting contracts with Hoffmann La-Roche, Switzerland, related to economic evaluations of Tamiflu for treatment and postexposure prophylaxis in epidemics and pandemics. This work involved giving presentations at scientific meetings, for which she received travel assistance and a speaker’s fee. David Fisman holds an Ontario Early Researcher Award funded by the Ontario Ministry of Research and Innovation. Matching funds for this grant were provided by Sanofi-Pasteur, which manufactures influenza vaccines, including vaccine for pH1N1 (for use outside Canada). None declared for Marija Zivkovic Gojovic, Murray Krahn and Chris Bauch.
